# Low Salivary Amylase Gene (*AMY1*) Copy Number Is Associated with Obesity and Gut *Prevotella* Abundance in Mexican Children and Adults

**DOI:** 10.3390/nu10111607

**Published:** 2018-11-01

**Authors:** Paola León-Mimila, Hugo Villamil-Ramírez, Blanca E. López-Contreras, Sofía Morán-Ramos, Luis R. Macias-Kauffer, Víctor Acuña-Alonzo, Blanca E. del Río-Navarro, Jorge Salmerón, Rafael Velazquez-Cruz, Teresa Villarreal-Molina, Carlos A. Aguilar-Salinas, Samuel Canizales-Quinteros

**Affiliations:** 1 Facultad de Química, Unidad de Genómica de Poblaciones Aplicada a la Salud, Universidad Nacional Autónoma de México (UNAM)/Instituto Nacional de Medicina Genómica (INMEGEN), Mexico City 14610, Mexico; paov_lemi@yahoo.com.mx (P.L.-M.); hugo_villamil@hotmail.com (H.V.-R.); blopez@inmegen.gob.mx (B.E.L.-C.); smoran@inmegen.gob.mx (S.M.-R.); luisrmacias@gmail.com (L.R.M.-K.); 2 Consejo Nacional de Ciencia y Tecnología (CONACYT), Mexico City 03940, Mexico; 3 Escuela Nacional de Antropología e Historia, Laboratorio de genética molecular, Mexico City 14030, Mexico; victor_acuna@inah.gob.mx; 4 Departamento de Alergia e Inmunología Clínica, Hospital Infantil de México Federico Gómez, Mexico City 06720, Mexico; blancadelrionavarro@gmail.com; 5 Unidad Académica de Investigación Epidemiológica del Centro de Investigación en Políticas, Población y Salud, Facultad de Medicina-UNAM, Mexico City 04510, Mexico; jorge.salmec@gmail.com; 6 Laboratorio de Genómica del Metabolismo Óseo, INMEGEN, Mexico City 14610, Mexico; rvelazquez@inmegen.gob.mx; 7 Laboratorio de Enfermedades Cardiovasculares, INMEGEN, Mexico City 14610, Mexico; mvillareal@inmegen.gob.mx; 8 Unidad de Investigación en Enfermedades Metabólicas and Departamento de Endocrinología y Metabolismo, Instituto Nacional de Ciencias Médicas y Nutrición Salvador Zubirán, Mexico City 14000, Mexico; caguilarsalinas@yahoo.com; 9 Tecnológico de Monterrey, Escuela de Medicina y Ciencias de la Salud, Monterrey, Nuevo León 64710, Mexico

**Keywords:** CNVs, obesity, Mexican, *AMY1*, *Prevotella*

## Abstract

Genome-wide association studies (GWAS) have identified copy number variants (CNVs) associated with obesity in chromosomal regions 1p31.1, 10q11.22, 11q11, 16p12.3, and recently 1p21.1, which contains the salivary amylase gene (*AMY1*). Recent evidence suggests this enzyme may influence gut microbiota composition through carbohydrate (mainly starch) degradation. The role of these CNVs in obesity has been scarcely explored in the Latino population, and thus the aim of our study was to evaluate the association of 1p31.1, 10q11.22, 11q11, 16p12.3 and 1p21.1 CNVs with obesity in 921 Mexican children, to replicate significant associations in 920 Mexican adults, and to analyze the association of *AMY1* copy number with gut microbiota in 75 children and 45 adults. Of the five CNVs analyzed, 1q11 CNV was significantly associated with obesity in children, but not in adults. Only *AMY1* CNV was significantly associated with obesity in both age groups. Moreover, gut microbiota analyses revealed a positive correlation between *AMY1* copy number and *Prevotella* abundance. This genus has enzymes and gene clusters essential for complex polysaccharide degradation and utilization. To our knowledge, this is the first study to analyze the association of these five CNVs in the Mexican population and to report a correlation between *AMY1* CN and gut microbiota in humans.

## 1. Introduction

Obesity represents a major global health problem [1]. In Mexico, according to the National Health and Nutrition Survey, the prevalence of overweight and obesity is 33.2% in children and 72.5% in adults [2]. Childhood obesity is the main predictor of adulthood obesity, which is associated with a number of metabolic and cardiovascular risk factors including type 2 diabetes, dyslipidemia, and hypertension [3,4].

Although the high prevalence of obesity has been attributed to an obesogenic environment, obesity has high heritability estimates ranging from 40 to 80% [5]. To date, more than 90 single nucleotide polymorphism (SNPs) have been associated with body mass index (BMI) mainly in Caucasian populations [6,7,8,9,10,11], some of which were also associated with BMI in Mexican children and adults [12]. Altogether, these SNPs account for less of 5% of the variability of this trait [11,12]. It has been suggested that copy number variation (CNV) might contribute to explain the missing heritability of obesity [13]. In this regard, genome-wide association studies (GWAS) have revealed certain CNV regions associated with obesity in European and Asian populations, including chromosomal regions 1p31.1, 10q11.22 11q11, 16p12.3, and more recently 1p21.1 which includes the salivary amylase gene *AMY1* [9,10,14,15,16]. Interestingly, Falchi et al. reported that the effect size of the *AMY1* CNV on obesity risk was considerably higher than that of other genetic variants such as fat mass and obesity-associated (*FTO*) gene polymorphisms [16]. However, subsequent studies evaluating the association of *AMY1* with obesity have reported conflicting results [17,18].

Because *AMY1* CNV has been associated with obesity and is considered one of the strongest signals of recent selection on human populations [19], there is great interest in unraveling the role of *AMY1* in human obesity. *AMY1* copy number (CN) correlates positively with salivary amylase amount and activity [19,20,21], and could likely influence gut microbiota composition through dietary carbohydrate processing. In this regard, the *Prevotella*-driven enterotype appears to be predominant in subjects consuming high proportions of dietary carbohydrate and fiber [22,23], however, its relationship with *AMY1* CN has not been studied. Moreover, a study in mice reported an association of the *AMY1* locus with both weight gain and increased *Enterobacteria* relative abundance in the gut [24]. 

The role of CNVs in obesity has been scarcely explored in the Mexican population, and thus the aim of our study was to evaluate the association of five candidate copy number variants (1p31.1, 10q11.22, 11q11, 16p12.3 and 1p21.1) with obesity in Mexican children, to replicate significant associations in Mexican adults, and to analyze the association of *AMY1* copy number with gut genera and species belonging to *Enterobacteriaceae* and *Prevotellaceae* families and their role in obesity in Mexican children and adults.

## 2. Materials and Methods

### 2.1. Case-Control Studies in Children and Adults

A total of 921 unrelated Mexican mestizo children aged 6–12 years (485 normal weight controls and 436 obesity cases) were recruited from a summer camp for children of employees of the Mexican Health Ministry and Hospital Infantil de México. The adult cohort included 920 unrelated Mexican-Mestizo adults aged 18–75 years (536 with normal weight and 384 with obesity). Obese and normal-weight adults were recruited from several health institutions and public universities in Mexico City. Recruitment and inclusion criteria for children and adults have been described elsewhere [12,25]. All participants answered a detailed questionnaire providing demographic and lifestyle information. The study protocol was performed in accordance with the Declaration of Helsinki and was approved by the Ethic Committees of participant institutions. All adult participants and parents or legal guardians of children provided informed consent, and all children assented to participate.

### 2.2. Anthropometric and Biochemical Parameters

Anthropometric measurements including weight, height, waist, and hip circumference were determined following the procedures recommended by Lohman et al. [26]. BMI was calculated as body weight divided by height squared (kg/m^2^). For children, BMI percentile was calculated using age and sex-specific BMI reference data as recommended by the Centers for Disease Control and Prevention, and obesity was defined as BMI ≥95th percentile [27]. In adults, obesity status was determined according to World Health Organization (WHO) criteria [28]. Body composition was measured by bioelectrical impedance using a BIA (BIA 101 RJL System) body composition analyzer (RJL Systems, Clinton Township, MI, USA) only in children. Biochemical parameters including fasting total cholesterol (TC), triglyceride (TG), HDL-C (High Density Lipoprotein Cholesterol), glucose and insulin levels were performed using standardized procedures as previously described [12].

### 2.3. CNV Quantification

Genomic DNA was isolated from peripheral leukocytes using the QIAmp DNA Blood Mini Kit (QIAmp 96 DNA Blood Kit, Quiagen, Hilden, Germany). Copy number quantification was estimated by duplex quantitative real-time PCR (qPCR) with two TaqMan assays (Table S1), one for the target CNV and the other for the reference gene (*RNaseP*) (Life Technologies, Pleasanton, CA, USA). Assays were performed in a ViiA7 Real-Time PCR instrument (Thermo Fisher, Waltham, MA, USA). Relative copy number values were calculated by the ΔΔCT method using Copy Caller software (v.2.0, Applied Biosystems, Foster City, CA, USA), and results were validated only when calling confidence was >80% and ΔCq standard deviation between replicates was <0.20. All quantification assays were performed in triplicate. *AMY1* CNV quantification was verified by droplet digital PCR (ddPCR System, Bio-Rad). Reactions were performed following the manufacturer’s recommendations using ddPCR™ Supermix and Taqman probes. Droplets were generated in a QX100 droplet device and amplification was performed by a C1000 Touch PCR thermal cycler (BioRad, Hercules, CA, USA). ddPCR data were analyzed using QuantaSoft software version 1.3.1.0. Four control samples obtained from the Coriell Institute for Medical Research were previously quantified and validated by Fiber-FISH [NA11930 (2 *AMY1* copies), NA10852 (6 *AMY1* copies), NA11993 (10 *AMY1* copies) and NA18972 (18 *AMY1* copies)] [29] were included as references to quantify *AMY1* copy number. We further validated *AMY1* CNs of these samples by digital PCR.

### 2.4. Gut Microbiota Analyses

Fecal samples were obtained from 75 children and 45 adult participants and DNA was extracted from these samples as previously described [30]. The V4 hypervariable region was amplified using 515F and 806R primers [31] and sequenced in an Illumina MiSeq 2 × 250 device. Sequences were analyzed using QIIME 1.9.1 [32], and phylogenetic distances were calculated by the UniFrac method [33]. A full description of 16S rRNA sequencing analyses has been described elsewhere [30]. Abundances were normalized using an arcsin transformation.

### 2.5. Dietary Assessment

A semi-quantitative food frequency questionnaire previously validated for the Mexican population [34] was applied to children and adults with available fecal samples. Average daily energy and nutrient intake was computed through the Evaluation System of Nutritional Habits and Nutrient Intake Software, and was expressed both as grams and percentage of total energy.

### 2.6. Statistical Analyses

Statistical analyses were performed with SPSS software version 18.0. Anthropometric and biochemical parameters in obese and normal-weight subjects were compared using the Student’s *t*-test or Mann-Whitney U-test. Associations of 1p31.1, 10q11.22, 11q11 and 16p12.3 CNVs with obesity were tested comparing the frequency of deletions (<2 copies) and duplications (>2 copies) in normal-weight and obese children. Given the high range of *AMY1* copy numbers (2–19), two different cutoff points were used to compare the copy number in normal and obese subjects: The *AMY1* CN median (CN < 6 vs. CN > 6), and the cutoff point used by Falchi et al. (CN ≤ 4 or low vs. CN ≥ 10 or high) [16]. All associations were tested by logistic regression, adjusting for age and sex. In addition, lineal regression models were used to test associations of CNVs with anthropometric and metabolic parameters adjusting for age, sex, and BMI as appropriate. Correlations between microbial relative abundance (genera and species belonging to *Enterobacteriaceae* and *Prevotellaceae* families) and *AMY1* CN or diet were evaluated using Spearman’s tests. Hochberg false discovery rate (FDR)-adjusted *q*-values < 0.05 were considered significant for the entire microbiota analysis [35]. Relative abundance of genera and species belonging to *Enterobacteriaceae* and *Prevotellaceae* families in low and high *AMY1* CN carriers was compared using the Mann-Whitney U-test.

## 3. Results

### 3.1. Clinical Characteristics of Case-Control Study

Clinical and biochemical characteristics of 921 children and 920 adults stratified by nutritional status are shown in Table S2. As expected, normal-weight children and adults had significantly lower obesity-related anthropometric measurements and lower biochemical measurements (insulin, HOMA-IR (Homeostatic Model Assessment for Insulin Resistance), triglycerides and total cholesterol levels) as compared to children and adults with obesity (*P* ≤ 0.01).

### 3.2. Association of 11q11and 1p21.1 (AMY1) CNVs with Obesity in Mexican Children

**Figure 1:**
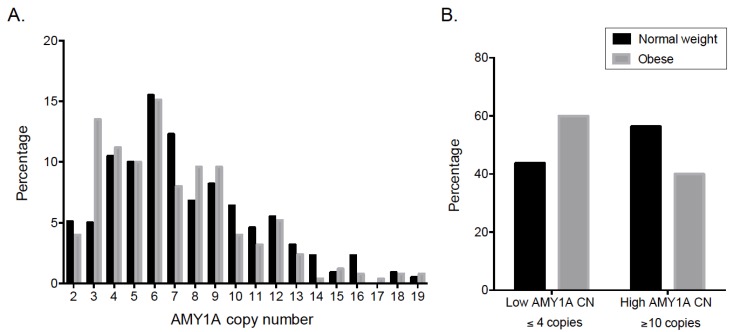
Distribution of *AMY1* copy number in normal weight and obese children (*n* = 921). (**A**) *AMY1* copy numbers ranged from 2 to 19. Normal-weight children are represented by gray bars and obese children by black bars; (**B**) Distribution of low vs high *AMY1* copy numbers in normal weight and obese children. Low *AMY1* copy numbers were significantly more frequent in obese than in normal weight children (*P* = 0.035).

The associations of the five CNV loci with obesity in children are shown in Table 1. Copy numbers in loci 16p12.3, 1p31.1 and 10q11.22 ranged from 1 to 4 in Mexican children. Deletions within these loci were infrequent (≤5%), and were not significantly associated with obesity (*P* ≥ 0.1). In contrast, 11q11/*OR4P4/OR4S2/OR4C6* (olfactory receptors family) and 1p21.1/*AMY1* CNVs ranged from 0 to 8 and from 2 to 19, respectively. Heterozygous or homozygous deletions of *OR4P4/OR4S2/OR4C6* CNV were significantly associated with lower obesity risk (OR = 0.774; 95% CI = 0.634–0.945; *P =* 0.047). The distribution of *AMY1* copy numbers in obese and normal weight children is shown in Figure 1A (median CN = 6). Children with <6 *AMY1* copies had a borderline significantly higher risk of obesity as compared to those with ≥6 *AMY1* CNs (OR = 1.323; 95% CI = 0.984–1.780, *P =* 0.064) (Table 1). Moreover, using the previously reported cutoff values of ≤4 *AMY1* copies (low) and ≥10 *AMY1* copies (high), low *AMY1* copy numbers were significantly associated with obesity (OR = 1.530; 95% CI = 1.030–2.273, *P =* 0.035; after adjusting for age and gender) (Figure 1B). No significant associations between 11q11/*OR4P4/OR4S2/OR4C6* or 1p21.1/*AMY1* CNVs and biochemical traits were observed in children (Table S3).

### 3.3. Association of AMY1 Copy Number with Obesity in Mexican Adults

We then sought whether the associations found in children were also observed in Mexican adults. The 11q11 CNV was not associated with obesity in adults (*P =* 0.537). However, the presence of less than six *AMY1* copies showed a borderline significant association with obesity (OR = 1.521; 95% CI = 0.928–2.495; *P* = 0.096), and individuals with a low number of *AMY1* copies (≤4) had a significantly higher risk of obesity than those with a high number (≥10) of *AMY1* copies (OR = 1.536; 95% CI = 1.019–2.313, *P* = 0.040) (Table 2). As observed in children, *AMY1* CNV showed no significant associations with biochemical parameters (Table S4).

### 3.4. Association of AMY1 Copy Number with Prevotella Abundance

**Figure 2:**
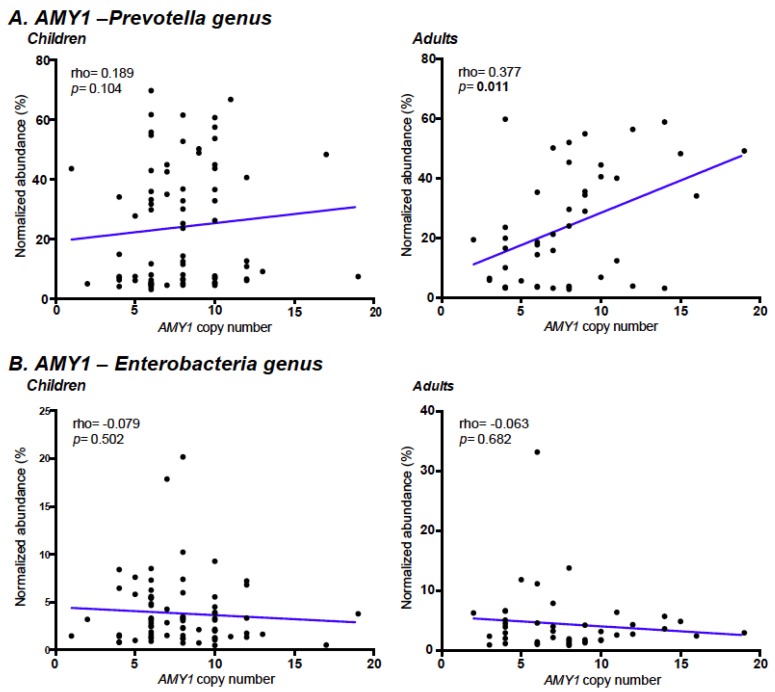
Correlations of *AMY1* CN with relative abundance of *Prevotella* and *Enterobacteria* genera in Mexican individuals. Bacterial abundances of *Prevotella* (**A**) and *Enterobacteria* (**B**) were normalized using arcsin sqrt transformation, and Spearman’s correlation coefficients were estimated.

Correlations between *AMY1* copies and the relative abundance of *Prevotella* and *Enterobacteria* genera in both children and adults are shown in Figure 2. No significant correlations between *Enterobacteria* and *AMY1* copy number were observed in any age group (Figure 2B). However, a positive and significant correlation between *AMY1* copy number and *Prevotella* abundance was observed in adults (r = 0.377; *P* = 0.011), while a positive correlation that did not reach statistical significance was observed in children (r = 0.189; *P* = 0.104) (Figure 2A). We then explored correlations between *AMY1* CN and all other identified gut microbiota genera. In addition to the relative abundance of *Prevotella* that showed a positive and significant correlation in adults, no other taxa were significantly associated with *AMY1* CN (Table S5).

**Figure 3:**
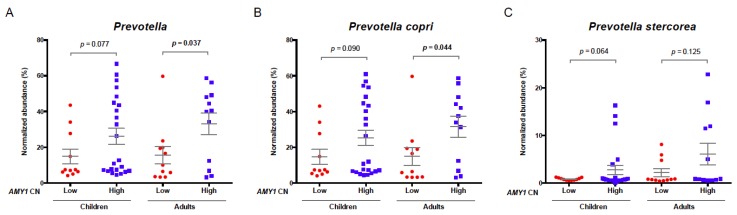
Comparison of *Prevotella* relative abundance in Mexican individuals with low vs. high *AMY1* CNs. Average abundances of *Prevotella* (**A**), *Prevotella copri* (**B**) and *Prevotella stercorea* (**C**) were higher in both children and adults with high (≥10) as compared to low (≤4 copies) *AMY1* copy numbers. Differences reached statistical significance only for *Prevotella genus* and *Prevotella copri* in adults.

Moreover, the relative abundance of *Prevotella*, specifically of *Prevotella copri*, was twofold higher in adults with a high number of *AMY1* copies (≥10) as compared to those with a low *AMY1* copy number (≤4). These comparisons were similar in children, without reaching statistical significance (*P* = 0.077 and 0.090, respectively; adjusted by age, gender and BMI percentile) (Figure 3; Table S6).

We then compared dietary carbohydrate intake in individuals with low and high *AMY1* CN, observing no differences in the consumption of any carbohydrate (Table S7). Moreover, no significant correlations were observed between *Prevotella* genus or species abundance with dietary carbohydrates, fiber or starch intake (*P* > 0.05) (Figure S1).

## 4. Discussion

Association studies of copy number variants with obesity are limited and have reported inconsistent results [9,10,14,15,16,17,18]. In the present study, we sought associations of five obesity-related CNV loci [1p31.1/*NEGR1* (Neuronal growth regulator 1), 10q11.22/*GPRC5B* (G Protein-Coupled Receptor Class C Group 5 Member B), 11q11/*OR4P4/OR4S2/OR4C6*, 16p12.3/*NPY4R* (Neuropeptide Y Receptor Y4) and 1p21.1/*AMY1*] with obesity risk in the Mexican population. In contrast with previous findings, *NEGR1*, *GPRC5* and *NPY4R* CNVs were not associated with obesity in Mexican children. As with all association studies, this could be due to an array of factors including ethnic, study design and methodological differences, or gene-environment interactions, among others [36,37]. The lack of association of the 1p31.1/*NEGR1* CNV is in agreement with previous reports in Mexican children and adults where rs2815752 (in high LD with 1p31.1/*NEGR1* CNV) was not associated with obesity [12,38], but in disagreement with the findings of Antúnez-Ortiz et al. who found a significant association of another *NEGR1* CNV in a group of Mexican children [39]. Regarding the *GPCR5* CNV, it has been previously suggested that the effect of the deletion of CNV is ethnic-specific, as it was significantly associated with obesity in Europeans, who have a higher CNV deletion frequency (0.27); but not in the Chinese population who have a lower CNV deletion frequency (0.008) [40]. The *GPCR5* CNV deletion frequency was 0.025 in the Mexican population, and was not associated with obesity, which is consistent with the previous findings in Chinese individuals. Finally, previous studies associating the *NPY4R* CNV with obesity risk have reported contradictory findings, as the CNV deletion allele has been associated both with higher and lower obesity risk [14,15,41,42]. Our findings are consistent with the report of Sun et al., who found no association of this CNV deletion with obesity in the Chinese population [43].

Interestingly, 11q11/*OR4P4/OR4S2/OR4C6* and 1p21.1/*AMY1* CNVs were significantly associated with obesity in Mexican children. The association of the *OR4P4/OR4S2/OR4C6* CNV is consistent with the previously reported role of olfactory receptor gene polymorphisms in obesity [44], and the role of the olfactory system in thermogenesis and energy homeostasis in the murine model [45]. However, while the 11q11 CNV deletion was associated with a decreased risk of obesity in Mexican children, previous studies have reported this CNV deletion is associated with increased obesity risk [15,46]. Moreover, the 11q11 CNV deletion was not associated with obesity in Mexican adults. These age-dependent association differences could be due to higher BMI heritability in childhood [47], higher effect sizes of obesity-associated loci in childhood, or to age-dependent gene–environment interactions [48,49].

The role of *AMY1* copy number variation in obesity risk has been widely studied [16,50,51,52], with inconsistent results [17,18]. It has been suggested that *AMY1* CN variation among human populations may result from natural selection; however, starch digestion does not seem to be the major selective force [53]. In this study, we did not observe *AMY1* CN differences between Mexican Mestizo and Indigenous subjects (mean 7.0 ± 3.2 in Mexican-Mestizos and 7.2 ± 3.7 in 130 Mexican indigenous; *P* = 0.511). This is in agreement with a recent report suggesting that a high number of *AMY1* copies were fixed in modern human populations, after the separation of humans and Neanderthals [54].

Interestingly, *AMY1* was the only loci associated with obesity in both children and adults. Falchi et al. first reported a remarkably strong association of low *AMY1* copy numbers with increased obesity risk in Europeans [16]. Further studies replicated this association in other populations and in different age groups, although with considerably lower effect sizes [50,51,52]. A previous study in Mexican children reported an association of *AMY1* copy numbers with obesity [55], although Usher et al. [17] suggested that this study described an outlier set of control samples with unusually high *AMY1* copy number measurements, which is not necessarily a replication of earlier findings. In the present study, we observed the initially reported shifted distribution of *AMY1* copy number between individuals with normal weight and obesity. Altogether, these findings confirm the role of this CNV in obesity in Mexican children and adults.

The mechanism by which *AMY1* copy number plays a role in obesity remains unclear. Our study evaluated the *AMY1* CN-gut microbiota-obesity association in a subgroup of Mexican children and adults. We did not observe significant correlations between *AMY1* copy number and *Enterobacteriaceae* family (Table S6), as previously reported in the murine model [24]. However, the gut microbiome of children and adults with high *AMY1* copy numbers was enriched in *Prevotella* genus and species, but not with any other gut microbiota genera, stressing the importance of further studies on the possible role of *AMY1* function on this bacterial genus. *Prevotella* is one of the most abundant enterotypes in the intestinal microbiome and has enzymes and gene clusters essential for fermentation and utilization of complex polysaccharides [22,23,56]. This is consistent with results of several studies showing that increased *Prevotella* is metabolically favorable [23], and that a high *Prevotella*/*Bacteroides* ratio favors weight loss in response to certain dietary interventions [57,58]. However, while we observed significant associations of high *AMY1* CN with both normal weight and increased *Prevotella* abundance (specifically *Prevotella copri*), *Prevotella* abundance was not significantly associated with nutritional status or with total dietary carbohydrates (including fiber and starch). Further studies using larger sample sizes will define whether this lack of association is due to insufficient statistical power.

This study has certain limitations that should be acknowledged. Firstly, the sample size was insufficient to detect associations of obesity with low frequency alleles (1p31.1/*NEGR1*, 10q11.22/*GPRC5B*, and 16p12.3/*NPY4R*), although it was sufficient to find associations with 11q11/*OR4P4/OR4S2/OR4C6*, and 1p21.1/*AMY1* CNs. Secondly, although amylase activity is clearly associated with obesity [19,20,21], this parameter was not measured in the present study. Finally, intestinal microbiome analysis was performed in a small number of individuals, limiting statistical power to associate the entire intestinal microbiome with *AMY1* CNs. However, the sample size was sufficient to find a positive correlation between *Prevotella* abundance and *AMY1* CNs.

## 5. Conclusions

In conclusion, of the five CNVs previously associated with obesity, only *AMY1* CNV was significantly associated with obesity in Mexican children and adults. Moreover, gut microbiota analyses identified a positive correlation between *AMY1* copy number and *Prevotella* abundance, which highlights the role of genetics in the modulation of intestinal microbiota. To our knowledge, this is the first study to report the association of *AMY1* CN and gut microbiota in humans. Future studies are required to identify mechanisms explaining these associations.

## Figures and Tables

**Table 1:** Association of five copy number variants with obesity risk in Mexican children (*n* = 921).

Locus	Gene	CN Range	Classification	Normal Weight		Obese		Association		
				*n*	%	*n*	%	OR	95% CI	*P*-value
11q11	*OR4P4*, *OR4S2*	0–8	Deletion	199	41.0	151	34.6	0.774	0.634–0.945	0.047
	*OR4C6*		≥2 copies	286	59.0	285	65.4			
1p21.1	*AMY1*	2–19	Less 6 copies	116	23.9	128	29.4	1.323	0.984–1.780	0.064
			≥6 copies	369	76.1	308	70.6			
16p12.3	*GPRC5B*	1–4	Deletion	11	2.3	13	2.9	1.307	0.472–3.622	0.606
			≥2 copies	474	97.7	423	97.1			
1p31.1	*NEGR1*	1–4	Deletion	25	5.2	23	5.3	1.078	0.425–3.622	0.867
			≥2 copies	460	94.8	413	94.7			
10q11.22	*NPY4R*	1–4	Deletion	21	4.3	19	4.4	1.064	0.411–2.758	0.898
			≥2 copies	464	95.7	417	95.6			

*OR, odds ratio; CI, confidence interval; OR4P4*, olfactory receptor family 4 subfamily P member 4 gene; *OR4S2*, olfactory receptor family 4 subfamily S member 2 gene; *OR4C6*, Olfactory receptor family 4 subfamily C member 6 gene; *AMY1*, salivary amylase gene; *GPRC5B*, G protein-coupled receptor class C group 5 member B gene; *NEGR1*, neuronal growth regulator 1 gene; *NPY4R*, neuropeptide Y receptor Y4 gene. Associations were tested by logistic regression and adjusted by sex and age.

**Table 2:** Association of 11q11 and 1p21.1 copy number variants with obesity risk in Mexican adults (*n* = 920).

Locus	Gene	CN Range	Classification	Normal Weight		Obese		Association		
				*n*	%	*n*	%	OR	95% CI	*P*-value
11q11	*OR4P4*, *OR4S2*	0–8	Deletion	124	32.3	186	34.7	1.054	0.877–1.266	0.537
	*OR4C6*		≥2 copies	260	67.7	350	65.3			
1p21.1	*AMY1*	2–19	Less 6 copies	94	24.5	165	30.8	1.521	0.928–2.495	0.096
			≥6 copies	290	75.5	371	69.2			

*OR, odds ratio; CI, confidence interval; OR4P4*, olfactory receptor family 4 subfamily P member 4 gene; *OR4S2*, olfactory receptor family 4 subfamily S member 2 gene; *OR4C6*, Olfactory receptor family 4 subfamily C member 6 gene; *AMY1*, salivary amylase gene. Associations were tested by logistic regression and adjusted by sex and age.

## Supplementary Materials

The following are available online at http://www.mdpi.com/2072-6643/10/11/1607/s1, Figure S1: Correlations of *Prevotella and Enterobacteria* abundances with *AMY1* CN, metabolic parameters and dietary carbohydrate intake. The correlation matrix depicts positive correlations in red and negative correlations in blue. The color scale indicates estimated Spearman’s correlation coefficient values. Statistical significance of each correlation is proportional to the size of the circle, Table S1: CNVs and assay designs used to test associations, Table S2: Clinical, anthropometric and biochemical parameters of the study population, Table S3: Association of 11q11 (*OR4P4/OR4S2/OR4C6*) and 1p21.1 (*AMY1*) copy number with biochemical parameters in Mexican children stratified by nutritional status, Table S4: Association of 11q11 and 1p21.1 copy number with biochemical parameters in Mexican adults stratified by nutritional status, Table S5: Correlations of *AMY1* CN with relative abundances of gut microbiota at the genus level in Mexican children and adults, Table S6: Comparison of normalized *Prevotellaceae* and *Enterobacteriaceae* abundances in Mexican individuals with low and high *AMY1* copy numbers, Table S7: Comparison of energy and dietary carbohydrate intake in Mexican individuals with low and high *AMY1* copy numbers.

## References

[1] Arroyo-Johnson C., Mincey K.D. (2016). Obesity Epidemiology Worldwide. Gastroenterol. Clin. N. Am..

[2] Shamah-Levy T., Rivera-Dommarco J., Hernández-Ávila M. (2016). Encuesta Nacional de Salud y Nutrición de Medio Camino.

[3] Freedman D.S., Khan L.K., Serdula M.K., Dietz W.H., Srinivasan S.R., Berenson G.S. (2005). The relation of childhood BMI to adult adiposity: The Bogalusa Heart Study. Pediatrics.

[4] Juonala M., Magnussen C.G., Berenson G.S., Venn A., Burns T.L., Sabin M.A., Srinivasan S.R., Daniels S.R., Davis P.H., Chen W. (2011). Childhood adiposity, adult adiposity, and cardiovascular risk factors. N. Engl. J. Med..

[5] Elks C.E., den Hoed M., Zhao J.H., Sharp S.J., Wareham N.J., Loos R.J., Ong K.K. (2012). Variability in the heritability of body mass index: A systematic review and meta-regression. Front. Endocrinol..

[6] Frayling T.M., Timpson N.J., Weedon M.N., Zeggini E., Freathy R.M., Lindgren C.M., Perry J.R., Elliott K.S., Lango H., Rayner N.W. (2007). A common variant in the *FTO* gene is associated with body mass index and predisposes to childhood and adult obesity. Science.

[7] Loos R.J., Lindgren C.M., Li S., Wheeler E., Zhao J.H., Prokopenko I., Inouye M., Freathy R.M., Attwood A.P., Beckmann J.S. (2008). Common variants near *MC4R* are associated with fat mass, weight and risk of obesity. Nat. Genet..

[8] Thorleifsson G., Walters G.B., Gudbjartsson D.F., Steinthorsdottir V., Sulem P., Helgadottir A., Styrkarsdottir U., Gretarsdottir S., Thorlacius S., Jonsdottir I. (2009). Genome-wide association yields new sequence variants at seven loci that associate with measures of obesity. Nat. Genet..

[9] Willer C.J., Speliotes E.K., Loos R.J., Li S., Lindgren C.M., Heid I.M., Berndt S.I., Elliott A.L., Jackson A.U., Lamina C. (2009). Six new loci associated with body mass index highlight a neuronal influence on body weight regulation. Nat. Genet..

[10] Speliotes E.K., Willer C.J., Berndt S.I., Monda K.L., Thorleifsson G., Jackson A.U., Lango Allen H., Lindgren C.M., Luan J., Magi R. (2010). Association analyses of 249,796 individuals reveal 18 new loci associated with body mass index. Nat. Genet..

[11] Locke A.E., Kahali B., Berndt S.I., Justice A.E., Pers T.H., Day F.R., Powell C., Vedantam S., Buchkovich M.L., Yang J. (2015). Genetic studies of body mass index yield new insights for obesity biology. Nature.

[12] Leon-Mimila P., Villamil-Ramirez H., Villalobos-Comparan M., Villarreal-Molina T., Romero-Hidalgo S., Lopez-Contreras B., Gutierrez-Vidal R., Vega-Badillo J., Jacobo-Albavera L., Posadas-Romeros C. (2013). Contribution of common genetic variants to obesity and obesity-related traits in mexican children and adults. PLoS ONE.

[13] Manolio T.A., Collins F.S., Cox N.J., Goldstein D.B., Hindorff L.A., Hunter D.J., McCarthy M.I., Ramos E.M., Cardon L.R., Chakravarti A. (2009). Finding the missing heritability of complex diseases. Nature.

[14] Sha B.Y., Yang T.L., Zhao L.J., Chen X.D., Guo Y., Chen Y., Pan F., Zhang Z.X., Dong S.S., Xu X.H. (2009). Genome-wide association study suggested copy number variation may be associated with body mass index in the Chinese population. J. Hum. Genet..

[15] Jarick I., Vogel C.I., Scherag S., Schafer H., Hebebrand J., Hinney A., Scherag A. (2011). Novel common copy number variation for early onset extreme obesity on chromosome 11q11 identified by a genome-wide analysis. Hum. Mol. Genet..

[16] Falchi M., El-Sayed Moustafa J.S., Takousis P., Pesce F., Bonnefond A., Andersson-Assarsson J.C., Sudmant P.H., Dorajoo R., Al-Shafai M.N., Bottolo L. (2014). Low copy number of the salivary amylase gene predisposes to obesity. Nat. Genet..

[17] Usher C.L., Handsaker R.E., Esko T., Tuke M.A., Weedon M.N., Hastie A.R., Cao H., Moon J.E., Kashin S., Fuchsberger C. (2015). Structural forms of the human amylase locus and their relationships to SNPs, haplotypes and obesity. Nat. Genet..

[18] Yong R.Y., Mustaffa S.B., Wasan P.S., Sheng L., Marshall C.R., Scherer S.W., Teo Y.Y., Yap E.P. (2016). Complex Copy Number Variation of *AMY1* does not Associate with Obesity in two East Asian Cohorts. Hum. Mutat..

[19] Perry G.H., Dominy N.J., Claw K.G., Lee A.S., Fiegler H., Redon R., Werner J., Villanea F.A., Mountain J.L., Misra R. (2007). Diet and the evolution of human amylase gene copy number variation. Nat. Genet..

[20] Mandel A.L., Peyrot des Gachons C., Plank K.L., Alarcon S., Breslin P.A. (2010). Individual differences in *AMY1* gene copy number, salivary alpha-amylase levels, and the perception of oral starch. PLoS ONE.

[21] Yang Z.M., Lin J., Chen L.H., Zhang M., Chen W.W., Yang X.R. (2015). The roles of *AMY1* copies and protein expression in human salivary alpha-amylase activity. Physiol. Behav..

[22] Wu G.D., Chen J., Hoffmann C., Bittinger K., Chen Y.Y., Keilbaugh S.A., Bewtra M., Knights D., Walters W.A., Knight R. (2011). Linking long-term dietary patterns with gut microbial enterotypes. Science.

[23] Kovatcheva-Datchary P., Nilsson A., Akrami R., Lee Y.S., De Vadder F., Arora T., Hallen A., Martens E., Bjorck I., Backhed F. (2015). Dietary Fiber-Induced Improvement in Glucose Metabolism is Associated with Increased Abundance of *Prevotella*. Cell Metab..

[24] Parks B.W., Nam E., Org E., Kostem E., Norheim F., Hui S.T., Pan C., Civelek M., Rau C.D., Bennett B.J. (2013). Genetic control of obesity and gut microbiota composition in response to high-fat, high-sucrose diet in mice. Cell Metab..

[25] Villalobos-Comparan M., Teresa Flores-Dorantes M., Teresa Villarreal-Molina M., Rodriguez-Cruz M., Garcia-Ulloa A.C., Robles L., Huertas-Vazquez A., Saucedo-Villarreal N., Lopez-Alarcon M., Sanchez-Munoz F. (2008). The *FTO* gene is associated with adulthood obesity in the Mexican population. Obesity.

[26] Lohman T.G., Roche A.F., Martorell R. (1991). Anthropometric Standardization Reference Manual Abridged Edition.

[27] Kuczmarski R.J., Ogden C.L., Guo S.S., Grummer-Strawn L.M., Flegal K.M., Mei Z., Wei R., Curtin L.R., Roche A.F., Johnson C.L. (2002). 2000 CDC Growth Charts for the United States: Methods and development. Vital Health Stat. 11.

[28] World Health Organization (2000). Obesity: Preventing and Managing the Global Epidemic. Report of a WHO Consultation.

[29] Carpenter D., Dhar S., Mitchell L.M., Fu B., Tyson J., Shwan N.A., Yang F., Thomas M.G., Armour J.A. (2015). Obesity, starch digestion and amylase: Association between copy number variants at human salivary (*AMY1*) and pancreatic (*AMY2*) amylase genes. Hum. Mol. Genet..

[30] Lopez-Contreras B.E., Moran-Ramos S., Villarruel-Vazquez R., Macias-Kauffer L., Villamil-Ramirez H., Leon-Mimila P., Vega-Badillo J., Sanchez-Munoz F., Llanos-Moreno L.E., Canizalez-Roman A. (2018). Composition of gut microbiota in obese and normal-weight Mexican school-age children and its association with metabolic traits. Pediatr. Obes..

[31] Caporaso J.G., Lauber C.L., Walters W.A., Berg-Lyons D., Huntley J., Fierer N., Owens S.M., Betley J., Fraser L., Bauer M. (2012). Ultra-high-throughput microbial community analysis on the Illumina HiSeq and MiSeq platforms. ISME J..

[32] Caporaso J.G., Kuczynski J., Stombaugh J., Bittinger K., Bushman F.D., Costello E.K., Fierer N., Pena A.G., Goodrich J.K., Gordon J.I. (2010). QIIME allows analysis of high-throughput community sequencing data. Nat. Methods.

[33] Lozupone C.A., Stombaugh J., Gonzalez A., Ackermann G., Wendel D., Vazquez-Baeza Y., Jansson J.K., Gordon J.I., Knight R. (2013). Meta-analyses of studies of the human microbiota. Genome Res..

[34] Hernandez-Avila M., Romieu I., Parra S., Hernandez-Avila J., Madrigal H., Willett W. (1998). Validity and reproducibility of a food frequency questionnaire to assess dietary intake of women living in Mexico City. Salud Publica Mex..

[35] Benjamini Y., Drai D., Elmer G., Kafkafi N., Golani I. (2001). Controlling the false discovery rate in behavior genetics research. Behav. Brain Res..

[36] Stryjecki C., Alyass A., Meyre D. (2018). Ethnic and population differences in the genetic predisposition to human obesity. Obes. Rev..

[37] Huang T., Hu F.B. (2015). Gene-environment interactions and obesity: Recent developments and future directions. BMC Med. Genom..

[38] Abadi A., Peralta-Romero J., Suarez F., Gomez-Zamudio J., Burguete-Garcia A.I., Cruz M., Meyre D. (2016). Assessing the effects of 35 European-derived BMI-associated SNPs in Mexican children. Obesity.

[39] Antunez-Ortiz D.L., Flores-Alfaro E., Burguete-Garcia A.I., Bonnefond A., Peralta-Romero J., Froguel P., Espinoza-Rojo M., Cruz M. (2017). Copy Number Variations in Candidate Genes and Intergenic Regions Affect Body Mass Index and Abdominal Obesity in Mexican Children. Biomed. Res. Int..

[40] Yang T.L., Guo Y., Li S.M., Li S.K., Tian Q., Liu Y.J., Deng H.W. (2013). Ethnic differentiation of copy number variation on chromosome 16p12.3 for association with obesity phenotypes in European and Chinese populations. Int. J. Obes..

[41] Aerts E., Beckers S., Zegers D., Van Hoorenbeeck K., Massa G., Verrijken A., Verhulst S.L., Van Gaal L.F., Van Hul W. (2016). CNV analysis and mutation screening indicate an important role for the *NPY4R* gene in human obesity. Obesity.

[42] Shebanits K., Andersson-Assarsson J.C., Larsson I., Carlsson L.M.S., Feuk L., Larhammar D. (2018). Copy number of pancreatic polypeptide receptor gene *NPY4R* correlates with body mass index and waist circumference. PLoS ONE.

[43] Sun C., Cao M., Shi J., Li L., Miao L., Hong J., Cui B., Ning G. (2013). Copy number variations of obesity relevant loci associated with body mass index in young Chinese. Gene.

[44] Choquette A.C., Bouchard L., Drapeau V., Lemieux S., Tremblay A., Bouchard C., Vohl M.C., Perusse L. (2012). Association between olfactory receptor genes, eating behavior traits and adiposity: Results from the Quebec Family Study. Physiol. Behav..

[45] Riera C.E., Tsaousidou E., Halloran J., Follett P., Hahn O., Pereira M.M.A., Ruud L.E., Alber J., Tharp K., Anderson C.M. (2017). The Sense of Smell Impacts Metabolic Health and Obesity. Cell Metab..

[46] Zhang D., Li Z., Wang H., Yang M., Liang L., Fu J., Wang C., Ling J., Zhang Y., Zhang S. (2015). Interactions between obesity-related copy number variants and dietary behaviors in childhood obesity. Nutrients.

[47] Guo G., Liu H., Wang L., Shen H., Hu W. (2015). The Genome-Wide Influence on Human BMI Depends on Physical Activity, Life Course, and Historical Period. Demography.

[48] Meng X.R., Song J.Y., Ma J., Liu F.H., Shang X.R., Guo X.J., Wang H.J. (2014). Association study of childhood obesity with eight genetic variants recently identified by genome-wide association studies. Pediatr. Res..

[49] Felix J.F., Bradfield J.P., Monnereau C., van der Valk R.J., Stergiakouli E., Chesi A., Gaillard R., Feenstra B., Thiering E., Kreiner-Moller E. (2016). Genome-wide association analysis identifies three new susceptibility loci for childhood body mass index. Hum. Mol. Genet..

[50] Viljakainen H., Andersson-Assarsson J.C., Armenio M., Pekkinen M., Pettersson M., Valta H., Lipsanen-Nyman M., Makitie O., Lindstrand A. (2015). Low Copy Number of the *AMY1* Locus Is Associated with Early-Onset Female Obesity in Finland. PLoS ONE.

[51] Marcovecchio M.L., Florio R., Verginelli F., De Lellis L., Capelli C., Verzilli D., Chiarelli F., Mohn A., Cama A. (2016). Low *AMY1* Gene Copy Number Is Associated with Increased Body Mass Index in Prepubertal Boys. PLoS ONE.

[52] Bonnefond A., Yengo L., Dechaume A., Canouil M., Castelain M., Roger E., Allegaert F., Caiazzo R., Raverdy V., Pigeyre M. (2017). Relationship between salivary/pancreatic amylase and body mass index: A systems biology approach. BMC Med..

[53] Fernandez C.I., Wiley A.S. (2017). Rethinking the starch digestion hypothesis for *AMY1* copy number variation in humans. Am. J. Phys. Anthropol..

[54] Inchley C.E., Larbey C.D., Shwan N.A., Pagani L., Saag L., Antao T., Jacobs G., Hudjashov G., Metspalu E., Mitt M. (2016). Selective sweep on human amylase genes postdates the split with Neanderthals. Sci. Rep..

[55] Mejia-Benitez M.A., Bonnefond A., Yengo L., Huyvaert M., Dechaume A., Peralta-Romero J., Klunder-Klunder M., Garcia Mena J., El-Sayed Moustafa J.S., Falchi M. (2015). Beneficial effect of a high number of copies of salivary amylase *AMY1* gene on obesity risk in Mexican children. Diabetologia.

[56] De Filippo C., Cavalieri D., Di Paola M., Ramazzotti M., Poullet J.B., Massart S., Collini S., Pieraccini G., Lionetti P. (2010). Impact of diet in shaping gut microbiota revealed by a comparative study in children from Europe and rural Africa. Proc. Natl. Acad. Sci. USA.

[57] Hjorth M.F., Roager H.M., Larsen T.M., Poulsen S.K., Licht T.R., Bahl M.I., Zohar Y., Astrup A. (2018). Pre-treatment microbial *Prevotella*-to-*Bacteroides* ratio, determines body fat loss success during a 6-month randomized controlled diet intervention. Int. J. Obes..

[58] Hjorth M.F., Blaedel T., Bendtsen L.Q., Lorenzen J.K., Holm J.B., Kiilerich P., Roager H.M., Kristiansen K., Larsen L.H., Astrup A. (2018). *Prevotella*-to-*Bacteroides* ratio predicts body weight and fat loss success on 24-week diets varying in macronutrient composition and dietary fiber: Results from a post-hoc analysis. Int. J. Obes..

