# Supplementary material for: Low Salivary Amylase Gene (AMY1) Copy Number Is Associated with Obesity and Gut Prevotella Abundance in Mexican Children and Adults
Source: Nutrients. 2018 Nov 1;10(11):1607. doi: 10.3390/nu10111607 (PMC6266693; doi:10.3390/nu10111607)
Supplement: Supplementary file 1 [file nutrients-10-01607-s001.zip › nutrients-367488-supplementary/Fig S1. Correlations of Prevotella_Enterobacteria abundances with AMY1 CN.pdf]

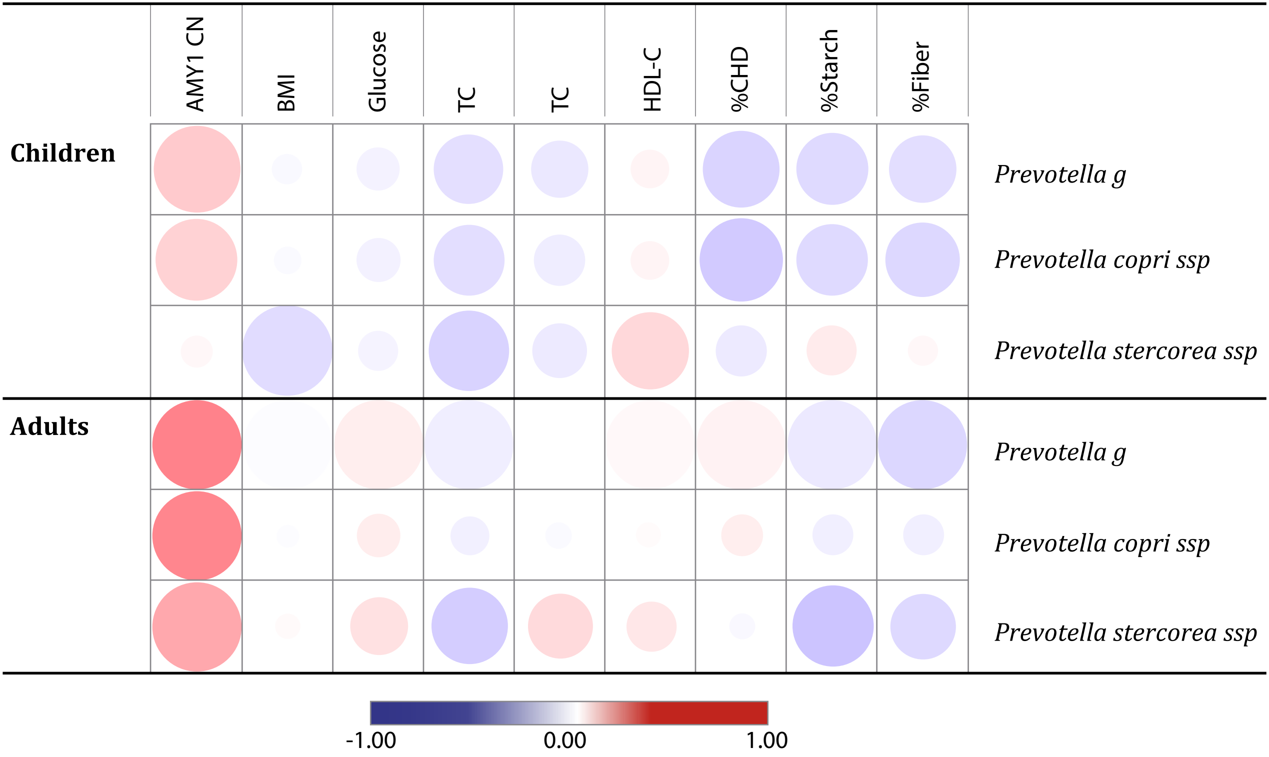

Fig S1. Correlations of *Prevotella/Enterobacteria* abundances with *AMY1* CN, metabolic parameters and dietary carbohydrate intake. The correlation matrix depicts positve correlations in red and negative correlation in blue. The color scale indicates estimated Pearson's correlation coefficient values. Statistical significance of each correlation is proportional to the size of the circle.
