# Supplementary material for: Low Salivary Amylase Gene (AMY1) Copy Number Is Associated with Obesity and Gut Prevotella Abundance in Mexican Children and Adults
Source: Nutrients. 2018 Nov 1;10(11):1607. doi: 10.3390/nu10111607 (PMC6266693; doi:10.3390/nu10111607)
Supplement: Supplementary file 1 [file nutrients-10-01607-s001.zip › nutrients-367488-supplementary/Table S1. CNVs and assay designs used to test associations.docx]

| **Table S1. CNVs and assay designs used to test associations.** | | | | | | | |
| --- | --- | --- | --- | --- | --- | --- | --- |
| **Region** | **CNV** | **Genes** | **Location (GRCh36)** | **Assay ID** | **Position (GRCh38)** | **Target genes** | **Reference** |
| 1p21.1 | 200-kb duplication | Covering *AMY1* | Chr1: 104198141 - 104301310 | Hs07226362_cn | Chr1:103655744 | 77 DGV IDs  *AMY1A*, *AMY1B* and *AMY1C* | Falchi *et al*, 2014. |
| 1p31.1 | 45-kb deletion | 20 kb upstream *NEGR1* | Chr1: 725,410,74-725,837,49 | Hs03098913_cn | Chr1:72332849 | 11 DGV IDs  *NEGR1* | Willer *et al*, 2009. |
| 10q11.22 | 474-kb deletion | Covering *NPY4R, SYT15*, *GPRIN2* | Chr10: 46,338,178–46,812,351 | Hs03742382_cn | Chr10:46546124 | 34 DGV IDs  *SYT15*, *GPRIN2,* *LOC728643* and *PPYR1* | Sha *et al*, 2009. |
| 11q11 | 80-kb deletion | *Covering OR4P4*, *OR4S2*, *OR4C6* | Chr11: 55,130,596-55,210,165 | Hs03802074_cn | Chr11:55679738 | 71 DGV IDs *OR4S2, OR4C6, OR4P4* | Jarick *et al*, 2011. |
| 16p12.3 | 21-kb deletion | 50 kb upstream *GPRC5B* | Chr16: 19,707,001-19,727,794 | Hs02931036_cn | Chr16:19858949 | 3 DGV IDs  *GPRC5B* | Spelliotes *et al*, 2010. |
| *CNV, copy number variant; Chr, chromosome; AMY1*, salivary amylase gene; *NEGR1*, neuronal growth regulator 1 gene; *NPY4R*, neuropeptide Y receptor Y4 gene; *SYT15*, Synaptotagmin 15 gene; *GPRIN2,* G protein regulated inducer of neurite outgrowth 2 gene; *OR4P4*, olfactory receptor family 4 subfamily P member 4 gene; *OR4S2*, olfactory receptor family 4 subfamily S member 2 gene; *OR4C6*, olfactory receptor family 4 subfamily C member 6 gene; *GPRC5B*, G protein-coupled receptor class C group 5 member B gene; Chr, chromosome. | | | | | | | |
