# Supplementary material for: Low Salivary Amylase Gene (AMY1) Copy Number Is Associated with Obesity and Gut Prevotella Abundance in Mexican Children and Adults
Source: Nutrients. 2018 Nov 1;10(11):1607. doi: 10.3390/nu10111607 (PMC6266693; doi:10.3390/nu10111607)
Supplement: Supplementary file 1 [file nutrients-10-01607-s001.zip › nutrients-367488-supplementary/Table S2. Clinical, anthropometric and biochemical parameters of the study population.docx]

| **Table S2. Clinical, anthropometric and biochemical parameters of the study population.** | | | | | | | | | | | | | | |
| --- | --- | --- | --- | --- | --- | --- | --- | --- | --- | --- | --- | --- | --- | --- |
|  |  | **Children (n=921)** | | | | | |  | **Adults (n=920)** | | | | | |
|  |  | **Normal weight** | |  | **Obese** | |  |  | **Normal weight** | |  | **Obese** | |  |
|  |  | (n=485) | |  | (n=436) | |  |  | (n=384) | |  | (n=536) | |  |
| **Trait** |  | n or median | % or IQR |  | n or median | % or IQR | ***P*** |  | n or median | % or IQR |  | n or median | % or IQR | ***P*** |
| Gender (Male) |  | 219 | 45.2 |  | 258 | 59.2 | <0.001 |  | 136 | 35.4 |  | 158 | 29.5 | 0.057 |
| Age (years) |  | 9.50 | 7.99-10.99 |  | 9.86 | 8.25-11.00 | 0.170 |  | 35.00 | 25.00-44.75 |  | 39.00 | 30.00-47.00 | 0.002 |
| BMI Percentile or BMI |  | 54.20 | 34.35-71.20 |  | 97.30 | 96.20-98.50 | <0.001 |  | 23.18 | 21.36-24.35 |  | 36.81 | 32.27-42.94 | <0.001 |
| Body weight (kg) |  | 29.00 | 24.05-37.00 |  | 49.05 | 39.00-58.00 | <0.001 |  | 59.00 | 54.00-65.00 |  | 97.95 | 82.49-115.93 | <0.001 |
| WC (cm) |  | 61.00 | 55.50-66.57 |  | 82.30 | 75.00-87.50 | <0.001 |  | 78.00 | 73.00-84.00 |  | 108.00 | 99.00-122.00 | <0.001 |
| WHR |  | 0.86 | 0.82-0.89 |  | 0.93 | 0.89-0.97 | <0.001 |  | 0.83 | 0.78-0.88 |  | 0.91 | 0.86-0.96 | <0.001 |
| Fat mass (%) |  | 25.10 | 20.20-29.85 |  | 44.10 | 40.78-48.03 | <0.001 |  | - | - |  | - | - | - |
| FG (mg/dL) |  | 91.00 | 85.00-96.00 |  | 91.10 | 86.00-95.00 | 0.878 |  | 91.00 | 84.00-97.00 |  | 96.00 | 89.00-107.00 | <0.001 |
| FI (mU/L) |  | 5.74 | 3.00-7.00 |  | 13.04 | 6.63-15.73 | <0.001 |  | 6.20 | 4.33-8.60 |  | 13.70 | 9.00-19.70 | <0.001 |
| HOMA-IR |  | 1.27 | 0.65-1.55 |  | 2.89 | 1.41-3.38 | <0.001 |  | 1.35 | 0.94-2.04 |  | 3.25 | 2.12-5.08 | <0.001 |
| TG (mg/dL) |  | 95.90 | 60.00-115.80 |  | 150.10 | 97.00-181.00 | <0.001 |  | 113.00 | 81.00-162.80 |  | 153.00 | 114.50-204.00 | <0.001 |
| TC (mg/dL) |  | 171.50 | 152.00-192.00 |  | 178.00 | 159.00-201.00 | <0.001 |  | 198.00 | 178.25-225.00 |  | 187.00 | 160.00-218.00 | 0.001 |
| HDL-C (mg/dL) |  | 51.00 | 44.00-59.00 |  | 41.00 | 35.00-48.00 | <0.001 |  | 50.00 | 42.00-58.00 |  | 38.00 | 33.00-45.00 | <0.001 |
| IQR, Interquartile range; BMI, Body mass index; WC, Waist circumference; WHR, Waist to hip ratio; FG, Fasting glucose; FI, Fasting insulin; HOMA-IR, homeostasis model assessment of insulin resistance; TG, Triglycerides; TC, Total cholesterol; HDL-C, High-density lipoprotein cholesterol.  Differences between groups were compared by X^2^, Student´s T test or Mann-Whitney’s U test.  Normal weight in children: BMI Percentile ≥15.0 ≤85.0, Obese: BMI Percentile ≥95.0; Normal weight in adults: BMI ≥18.5 ≤24.9, Obese: BMI ≥30.0. | | | | | | | | | | | | | | |
