# Supplementary material for: Low Salivary Amylase Gene (AMY1) Copy Number Is Associated with Obesity and Gut Prevotella Abundance in Mexican Children and Adults
Source: Nutrients. 2018 Nov 1;10(11):1607. doi: 10.3390/nu10111607 (PMC6266693; doi:10.3390/nu10111607)
Supplement: Supplementary file 1 [file nutrients-10-01607-s001.zip › nutrients-367488-supplementary/Table S3. Association of 11q11 (OR4P4:OR4S2:OR4C6) and 1p21.1 (AMY1) copy number with biochemical parameters in Mexican children stratified by nutritional status.docx]

| **Table S3. Association of 11q11 (*OR4P4/OR4S2/OR4C6*) and 1p21.1 (*AMY1*) copy number with biochemical parameters in Mexican children stratified by nutritional status.** | | | | | | | | | | | | | | | | | |
| --- | --- | --- | --- | --- | --- | --- | --- | --- | --- | --- | --- | --- | --- | --- | --- | --- | --- |
|  | |  | **11q11 (*OR4P4/OR4S2/OR4C6*) CNV** | | | | | | |  | **1p21.1 (*AMY1*) CNV** | | | | | | |
|  | |  | **Normal weight**  (n=485) | | |  | **Obese**  (n=436) | | |  | **Normal weight**  (n=485) | | |  | **Obese**  (n=436) | | |
| **Trait** | |  | B | SE | ***P*** |  | B | SE | ***P*** |  | B | SE | ***P*** |  | B | SE | ***P*** |
| FG (mg/dL) | |  | 0.271 | 0.193 | 0.155 |  | 0.149 | 0.197 | 0.396 |  | -0.217 | 0.130 | 0.104 |  | 0.207 | 0.117 | 0.092 |
| FI (µIU/mL) | |  | 0.061 | 0.093 | 0.772 |  | -0.107 | 0.342 | 0.579 |  | 0.025 | 0.065 | 0.650 |  | -0.358 | 0.234 | 0.181 |
| HOMA-IR | |  | 0.018 | 0.022 | 0.963 |  | -0.022 | 0.077 | 0.605 |  | 0.004 | 0.015 | 0.816 |  | -0.064 | 0.053 | 0.213 |
| TAG (mg/dL) | |  | -2.012 | 1.259 | 0.081 |  | -0.201 | 2.076 | 0.883 |  | 0.398 | 0.844 | 0.627 |  | -0.109 | 1.220 | 0.367 |
| TC (mg/dL) | |  | -0.396 | 0.696 | 0.570 |  | 0.473 | 0.872 | 0.588 |  | 0.910 | 0.468 | 0.053 |  | 0.238 | 0.512 | 0.643 |
| HDL-C (mg/dL) | |  | 0.011 | 0.261 | 0.966 |  | 0.013 | 0.261 | 0.959 |  | -0.016 | 0.177 | 0.930 |  | 0.212 | 0.153 | 0.166 |
| *OR4P4*, olfactory receptor family 4 subfamily P member 4 gene; *OR4S2*, olfactory receptor family 4 subfamily S member 2 gene; *OR4C6*, Olfactory receptor family 4 subfamily C member 6 gene; *AMY1*, salivary amylase gene; B, Beta; SE, Standard error; FG, Fasting glucose; FI, Fasting insulin; HOMA-IR, homeostasis model insulin resistance; TAG, Triglycerides; TC, Total cholesterol; HDL-C, high-density lipoprotein cholesterol.  Associations were tested by linear regression and adjusted by sex and age. | | | | | | | | | | | | | | | | | |
|  | | | | | | | | | | | | | | | | | |
