# Supplementary material for: Low Salivary Amylase Gene (AMY1) Copy Number Is Associated with Obesity and Gut Prevotella Abundance in Mexican Children and Adults
Source: Nutrients. 2018 Nov 1;10(11):1607. doi: 10.3390/nu10111607 (PMC6266693; doi:10.3390/nu10111607)
Supplement: Supplementary file 1 [file nutrients-10-01607-s001.zip › nutrients-367488-supplementary/Table S4. Association of 11q11 and 1p21.1 copy number with biochemical parameters in Mexican adults stratified by nutritional status.docx]

| **Table S4. Association of 11q11 and 1p21.1 copy number with biochemical parameters in Mexican adults stratified by nutritional status.** | | | | | | | | | | | | | | | | |
| --- | --- | --- | --- | --- | --- | --- | --- | --- | --- | --- | --- | --- | --- | --- | --- | --- |
|  |  | **11q11 (*OR4P4/OR4S2/OR4C6*) CNV** | | | | | | |  | **1p21.1 (*AMY1*) CNV** | | | | | | |
|  |  | **Normal weight**  (n=384) | | |  | **Obese**  (n=536) | | |  | **Normal weight**  (n=384) | | |  | **Obese**  (n=536) | | |
| **Trait** |  | B | SE | ***P*** |  | B | SE | ***P*** |  | B | SE | ***P*** |  | B | SE | ***P*** |
| FG (mg/dL) |  | 1.296 | 0.897 | 0.146 |  | -0.682 | 0.768 | 0.512 |  | 0.690 | 0.395 | 0.070 |  | -0.612 | 0.575 | 0.470 |
| FI (µIU/mL) |  | -0.052 | 0.122 | 0.698 |  | -0.041 | 0.310 | 0.875 |  | -0.001 | 0.063 | 0.739 |  | -0.125 | 0.203 | 0.962 |
| HOMA-IR |  | 0.001 | 0.036 | 0.797 |  | -0.047 | 0.120 | 0.942 |  | 0.013 | 0.018 | 0.404 |  | -0.067 | 0.082 | 0.827 |
| TG (mg/dL) |  | -3.607 | 4.198 | 0.240 |  | -1.270 | 2.750 | 0.615 |  | 0.788 | 1.886 | 0.999 |  | -1.751 | 1.610 | 0.404 |
| TC (mg/dL) |  | -0.990 | 1.121 | 0.378 |  | -0.465 | 0.939 | 0.620 |  | -0.276 | 0.542 | 0.611 |  | 0.275 | 0.563 | 0.626 |
| HDL-C (mg/dL) |  | 0.500 | 0.369 | 0.177 |  | 0.262 | 0.233 | 0.262 |  | 0.293 | 0.181 | 0.106 |  | 0.015 | 0.138 | 0.911 |
| *OR4P4*, *OR4S2*, *OR4C6*, Olfactory receptors family; *AMY1*, salivary amylase gene; B, Beta; SE, Standard error; FG, Fasting glucose; FI, Fasting insulin; HOMA-IR, homeostasis model insulin resistance; TG, Triglycerides; TC, Total cholesterol; HDL-C, high-density lipoprotein cholesterol.  Associations were tested by linear regression and adjusted by sex and age. | | | | | | | | | | | | | | | | |
