# Supplementary material for: Low Salivary Amylase Gene (AMY1) Copy Number Is Associated with Obesity and Gut Prevotella Abundance in Mexican Children and Adults
Source: Nutrients. 2018 Nov 1;10(11):1607. doi: 10.3390/nu10111607 (PMC6266693; doi:10.3390/nu10111607)
Supplement: Supplementary file 1 [file nutrients-10-01607-s001.zip › nutrients-367488-supplementary/Table S5. Correlations of AMY1A CN with relative abundances of gut microbiota at the genus level in Mexican children and adults.docx]

| **Table S5. Correlations of AMY1 CN with relative abundances of gut microbiota at the genus level in Mexican children and adults.** | | | | | | |
| --- | --- | --- | --- | --- | --- | --- |
|  |  | **Children (n=75)** | |  | **Adults (n=45)** | |
|  |  | Rho | P-value |  | Rho | P-value |
| Actinobacteria Bifidobacteriaceae *Bifidobacterium* |  | 0.021 | 0.855 |  | -0.046 | 0.762 |
| Bacteroidetes Bacteroidaceae *Bacteroides* |  | -0.102 | 0.384 |  | -0.183 | 0.230 |
| Bacteroidetes Porphyromonadaceae *Parabacteroides* |  | -0.022 | 0.850 |  | 0.011 | 0.943 |
| Bacteroidetes Prevotellaceae *Prevotella* |  | 0.189 | 0.104 |  | 0.377 | **0.011** |
| Bacteroidetes Unclassified Rikenellaceae |  | -0.042 | 0.720 |  | -0.103 | 0.499 |
| Bacteroidetes Unclassified S24-7 |  | -0.050 | 0.669 |  | 0.237 | 0.116 |
| Bacteroidetes Unclassified Barnesiellaceae |  | -0.086 | 0.465 |  | -0.131 | 0.392 |
| Bacteroidetes Odoribacteraceae *Odoribacter* |  | -0.007 | 0.952 |  | -0.002 | 0.990 |
| Bacteroidetes Paraprevotellaceae *Paraprevotella* |  | -0.154 | 0.187 |  | -0.279 | 0.063 |
| Bacteroidetes Paraprevotellaceae *Prevotella* |  | 0.071 | 0.548 |  | -0.104 | 0.497 |
| Unclassified Cyanobacteria |  | -0.085 | 0.469 |  | -0.092 | 0.546 |
| Unclassified Firmicutes |  | 0.022 | 0.850 |  | -0.161 | 0.292 |
| Firmicutes Unclassified Christensenellaceae |  | -0.199 | 0.086 |  | 0.001 | 0.994 |
| Firmicutes Clostridiaceae *Clostridium* |  | -0.053 | 0.650 |  | -0.009 | 0.952 |
| Firmicutes Unclassified Lachnospiraceae |  | 0.046 | 0.693 |  | -0.228 | 0.133 |
| Firmicutes Lachnospiraceae *Coprococcus* |  | 0.018 | 0.877 |  | 0.175 | 0.250 |
| Firmicutes Lachnospiraceae *Lachnospira* |  | 0.004 | 0.975 |  | -0.232 | 0.096 |
| Firmicutes Lachnospiraceae *Roseburia* |  | 0.039 | 0.738 |  | -0.278 | 0.064 |
| Firmicutes Lachnospiraceae *Lachnobacterium* |  | - | - |  | -0.079 | 0.605 |
| Firmicutes Lachnospiraceae *Dorea* |  | - | - |  | -0.116 | 0.449 |
| Firmicutes Lachnospiraceae *Anaerostipes* |  | 0.158 | 0.177 |  | - | - |
| Firmicutes Lachnospiraceae *Blautia* |  | 0.024 | 0.838 |  | - | - |
| Firmicutes Unclassified Peptostreptococcaceae |  | -0.033 | 0.779 |  | - | - |
| Firmicutes Unclassified Ruminococcaceae |  | 0.035 | 0.768 |  | -0.137 | 0.368 |
| Firmicutes Ruminococcaceae *Faecalibacterium* |  | 0.185 | 0.112 |  | -0.189 | 0.213 |
| Firmicutes Ruminococcaceae *Oscillospira* |  | -0.101 | 0.390 |  | 0.026 | 0.866 |
| Firmicutes Ruminococcaceae *Ruminococcus* |  | -0.071 | 0.544 |  | 0.138 | 0.365 |
| Firmicutes Unclassified Veillonellaceae |  | - | - |  | 0.156 | 0.307 |
| Firmicutes Veillonellaceae *Dialister* |  | -0.066 | 0.575 |  | 0.020 | 0.896 |
| Firmicutes Veillonellaceae *Phascolarctobacterium* |  | -0.031 | 0.792 |  | -0.107 | 0.486 |
| Firmicutes Unclassified Erysipelotrichaceae |  | 0.170 | 0.144 |  | -0.133 | 0.384 |
| Firmicutes Erysipelotrichaceae *Catenibacterium* |  | - | - |  | 0.241 | 0.111 |
| Firmicutes Erysipelotrichaceae *Eubacterium* |  | - | - |  | 0.208 | 0.171 |
| Proteobacteria Alcaligenaceae *Sutterella* |  | -0.026 | 0.824 |  | 0.006 | 0.969 |
| Proteobacteria Desulfovibrionaceae *Desulfovibrio* |  | -0.006 | 0.960 |  | -0.072 | 0.638 |
| Proteobacteria Succinivibrionaceae *Succinivibrio* |  | -0.067 | 0.571 |  | 0.031 | 0.840 |
| Proteobacteria Unclassified Enterobacteriaceae |  | -0.079 | 0.502 |  | -0.210 | 0.166 |
| Proteobacteria Enterobacteriaceae *Klebsiella* |  | - | - |  | 0.269 | 0.074 |
| Proteobacteria Moraxellaceae *Acinetobacter* |  | - | - |  | -0.055 | 0.722 |
| Proteobacteria Pasteurellaceae *Haemophilus* |  | 0.036 | 0.759 |  | - | - |
| Proteobacteria Unclassified Comamonadaceae |  | 0.059 | 0.612 |  | - | - |
| Tenericutes Unclassified Anaeroplasmataceae |  | - | - |  | -0.048 | 0.752 |
| Verrucomicrobia Verrucomicrobiaceae *Akkermansia* |  | -0.115 | 0.326 |  | -0.261 | 0.083 |
| Genus bacterial abundances were normalized using arcsin sqrt transformation, and Spearman’s correlation coefficients were estimated.  All q-values were >0.4 | | | | | | |
