# Supplementary material for: Low Salivary Amylase Gene (AMY1) Copy Number Is Associated with Obesity and Gut Prevotella Abundance in Mexican Children and Adults
Source: Nutrients. 2018 Nov 1;10(11):1607. doi: 10.3390/nu10111607 (PMC6266693; doi:10.3390/nu10111607)
Supplement: Supplementary file 1 [file nutrients-10-01607-s001.zip › nutrients-367488-supplementary/Table S6. Comparison of normalized Prevotellaceae and Enterobactericiae abundances in Mexican individuals with low and high AMY1 copy numbers.docx]

| **Table S6. Comparison of normalized *Prevotellaceae* and *Enterobactericiae* abundances in Mexican individuals with low and high *AMY1* copy numbers.** | | | | | | | | | | | | | | | | | | |
| --- | --- | --- | --- | --- | --- | --- | --- | --- | --- | --- | --- | --- | --- | --- | --- | --- | --- | --- |
|  |  | ***Children*** | | | | |  | ***Adults*** | | | | |  | ***All*** | | | | |
|  |  | **Low *AMY1*** | | **High *AMY1*** | |  |  | **Low *AMY1*** | | **High *AMY1*** | |  |  | **Low *AMY1*** | | **High *AMY1*** | |  |
|  |  | CN (n=11) | | CN (n=23) | |  |  | CN (n=11) | | CN (n=12) | |  |  | CN (n=22) | | CN (n=35) | |  |
|  |  | Mean | SD | Mean | SD | ***P*** |  | Mean | SD | Mean | SD | ***P*** |  | Mean | SD | Mean | SD | ***P*** |
| Normalized abundances * |  |  |  |  |  |  |  |  |  |  |  |  |  |  |  |  |  |  |
| ***Prevotellaceae* family** |  |  |  |  |  |  |  |  |  |  |  |  |  |  |  |  |  |  |
| *Prevotella* |  | 14.85 | 4.15 | 26.10 | 4.52 | 0.077 |  | 15.56 | 4.96 | 33.10 | 6.03 | 0.037 |  | 15.20 | 14.80 | 28.50 | 21.37 | 0.008 |
| *Prevotella copri* |  | 14.69 | 4.14 | 25.23 | 4.36 | 0.090 |  | 14.83 | 5.06 | 31.48 | 5.83 | 0.044 |  | 14.76 | 14.96 | 27.37 | 20.61 | 0.010 |
| *Prevotella stercorea* |  | 0.84 | 0.08 | 2.77 | 0.99 | 0.064 |  | 2.99 | 0.82 | 6.10 | 2.24 | 0.125 |  | 1.52 | 2.01 | 3.91 | 6.05 | 0.037 |
| ***Enterobacteriaceae* family** |  |  |  |  |  |  |  |  |  |  |  |  |  |  |  |  |  |  |
| *Enterobacteria* |  | 3.51 | 0.90 | 3.12 | 0.48 | 0.674 |  | 3.88 | 0.64 | 3.54 | 0.44 | 0.660 |  | 3.70 | 2.53 | 3.26 | 2.05 | 0.480 |
| *AMY1*, salivary amylase gene; SD, Standard deviation.  * Arcsin square-root-transformed microbial abundances.  Abundances between individuals with Low (≤4 copies) vs High (≥10 copies) AMY1A copy numbers were compared using a Mann-Whitney U-test. | | | | | | | | | | | | | | | | | | |
