# Supplementary material for: Low Salivary Amylase Gene (AMY1) Copy Number Is Associated with Obesity and Gut Prevotella Abundance in Mexican Children and Adults
Source: Nutrients. 2018 Nov 1;10(11):1607. doi: 10.3390/nu10111607 (PMC6266693; doi:10.3390/nu10111607)
Supplement: Supplementary file 1 [file nutrients-10-01607-s001.zip › nutrients-367488-supplementary/Table S7. Comparison of energy and dietary carbohydrate intake in Mexican individuals with low and high AMY1 copy numbers.docx]

| **Table S7. Comparison of energy and dietary carbohydrate intake in Mexican individuals with low and high *AMY1* copy numbers.** | | | | | | | | | | | | |
| --- | --- | --- | --- | --- | --- | --- | --- | --- | --- | --- | --- | --- |
|  |  | ***Children*** | | | | |  | ***Adults*** | | | | |
|  |  | **Low *AMY1* CN** | | **High *AMY1* CN** | |  |  | **Low *AMY1* CN** | | **High *AMY1* CN** | |  |
| **Nutrient** |  | (n=11) | | (n=23) | |  |  | (n=11) | | (n=12) | |  |
|  |  | Mean | SD | Mean | SD | ***P*** |  | Mean | SD | Mean | SD | ***P*** |
| Total kilocalories |  | 2055.65 | 762.78 | 2412.26 | 1186.57 | 0.380 |  | 2099.93 | 908.29 | 1971.31 | 875.22 | 0.733 |
| Total carbohydrates (gr) |  | 251.19 | 93.64 | 292.42 | 142.88 | 0.401 |  | 259.49 | 98.60 | 265.78 | 171.81 | 0.916 |
| Total carbohydrates (%) |  | 49.25 | 5.43 | 48.61 | 5.34 | 0.757 |  | 51.70 | 9.97 | 50.69 | 13.33 | 0.840 |
| *Simple carbohydrates* (% TCarb) | | | | | | | | | | | | |
| Glucose |  | 6.21 | 3.07 | 6.97 | 2.97 | 0.511 |  | 7.65 | 2.31 | 7.67 | 3.04 | 0.985 |
| Fructose |  | 8.82 | 3.42 | 9.41 | 3.84 | 0.674 |  | 9.89 | 2.77 | 10.56 | 4.34 | 0.668 |
| Sucrose |  | 14.25 | 3.84 | 13.37 | 3.22 | 0.508 |  | 14.63 | 5.09 | 13.33 | 2.60 | 0.444 |
| Lactose |  | 8.29 | 4.44 | 9.88 | 4.50 | 0.357 |  | 5.14 | 5.52 | 4.85 | 3.94 | 0.885 |
| Maltose |  | 0.49 | 0.12 | 0.57 | 0.11 | 0.098 |  | 0.49 | 0.13 | 0.58 | 0.17 | 0.148 |
| *Complex carbohydrates* (% TCarb) | | | | | | | | | | | | |
| Starch |  | 25.06 | 8.26 | 22.78 | 7.59 | 0.449 |  | 28.59 | 10.99 | 22.62 | 9.43 | 0.176 |
| Crude dietary fiber |  | 2.11 | 0.58 | 1.91 | 0.54 | 0.354 |  | 2.24 | 0.70 | 2.52 | 1.12 | 0.478 |
| Dietary fiber |  | 9.13 | 1.71 | 8.98 | 1.62 | 0.816 |  | 9.22 | 2.52 | 9.68 | 2.40 | 0.661 |
| Soluble dietary fiber |  | 2.65 | 0.46 | 2.68 | 0.48 | 0.834 |  | 2.63 | 0.81 | 2.60 | 0.61 | 0.9.17 |
| Insoluble dietary fiber |  | 5.09 | 1.47 | 4.83 | 1.24 | 0.609 |  | 5.06 | 1.57 | 5.84 | 1.98 | 0.313 |
| *AMY1*, salivary amylase gene; CN, copy number; SD, Standard deviation; TCarb, Total carbohydrates.  Differences between individuals with Low (≤4 copies) vs High (≥10 copies) AMY1A copy numbers were compared using a Mann-Whitney U-test. | | | | | | | | | | | | |
